# P68 Quantitative structure–activity relationship (QSAR) analysis of herpes simplex virus (HSV) thymidine kinase inhibitors: identifying key molecular determinants of antiviral activity

**DOI:** 10.1093/jacamr/dlag102.074

**Published:** 2026-06-26

**Authors:** Aser Waleed Mohamed Marzok

**Affiliations:** School of Health, Medicine and Life Sciences, University of Hertfordshire, College Lane, Hatfield AL10 9AB, UK

## Abstract

**Background:**

Herpes simplex virus (HSV) infections affect millions globally and remain a significant clinical burden [1]. HSV thymidine kinase is a key antiviral drug target; however, increasing resistance to existing therapies, such as acyclovir, underscores the need for novel inhibitors [2]. Quantitative structure–activity relationship (QSAR) modelling enables the identification of molecular features driving biological activity and supports rational antiviral drug design [3].

**Objectives:**

To develop a QSAR model to identify key molecular descriptors influencing inhibitory activity against HSV thymidine kinase and to guide optimization of antiviral compounds.

**Methods:**

Inhibitory activity data and physicochemical descriptors for 16 phenylguanine analogues were obtained from an HSV dataset. A Pearson correlation matrix was used to assess relationships between 10 QSAR descriptors and biological activity (log[1/IC₅₀]). Lipophilicity (π) and steric bulk (Sterimol B1) were selected based on strong correlation with activity and low intercorrelation. Descriptor values were standardized using z-score normalization prior to modelling. A multiple linear regression model was developed using a training set of 12 compounds, while 4 structurally diverse analogues were reserved as an external validation set. Model performance was evaluated using the coefficient of determination (R²), and predictive accuracy was assessed by comparing predicted and observed activity values. The validated model was subsequently applied to predict activity in additional untested analogues.

**Results:**

The QSAR model demonstrated good predictive performance, explaining 73.8% of the variance in inhibitory activity (R²=0.7383). Both lipophilicity and steric bulk contributed positively to activity, with lipophilicity identified as the most influential descriptor. External validation confirmed model reliability, with predicted values closely matching observed data (R²=0.7669) and deviations within acceptable limits. Application of the model to untested analogues identified phenylazo, phenylsulphonyl, and butylamino substituents as the most promising candidates, with predicted log(1/IC₅₀) values of 6.54, 6.25, and 6.16, respectively. These findings indicate that increasing hydrophobicity and appropriate steric complementarity enhance HSV thymidine kinase inhibition.

**Conclusions:**

This study demonstrates that lipophilicity and steric bulk are key determinants of HSV thymidine kinase inhibitory activity. The developed QSAR model provides a reliable and efficient tool for predicting antiviral activity and prioritizing compounds for synthesis. The findings support the role of hydrophobic interactions and steric optimization in ligand–enzyme binding and highlight the value of QSAR modelling in guiding early-stage antiviral drug discovery. This approach offers a cost-effective strategy for rational compound selection and optimization.
